# Health Economic Evaluation of Cognitive Control Training for Depression: Key Considerations

**DOI:** 10.2196/44679

**Published:** 2023-08-18

**Authors:** Constance Nève de Mévergnies, Nick Verhaeghe, Ernst H W Koster, Chris Baeken, Yannick Vander Zwalmen, Kristof Hoorelbeke

**Affiliations:** 1 Department of Experimental Clinical and Health Psychology Ghent University Ghent Belgium; 2 Department of Public Health and Primary Care Ghent University Ghent Belgium; 3 Department of Public Health Vrije Universiteit Brussel Brussels Belgium; 4 Department of Head and Skin Ghent University Ghent Belgium; 5 Department of Psychiatry University Hospital Brussel (UZBrussel) Brussels Belgium; 6 Department of Electrical Engineering Eindhoven University of Technology Eindhoven Netherlands

**Keywords:** health economic evaluation, cost utility, cognitive control training, CCT, depression recurrence, health policies

## Abstract

Depression is a serious and burdensome psychiatric illness that contributes heavily to health expenditures. These costs are partly related to the observation that depression is often not limited to a single episode but can recur or follow a chronic pathway. In terms of risk factors, it is acknowledged that cognitive impairments play a crucial role in vulnerability to depression. Within this context, cognitive control training (CCT) has shown its effectiveness in reducing the risk for recurrence of depression. CCT is low cost intensive and can be provided as a web-based intervention, which makes it easy to disseminate. Despite increasing interest in the field, studies examining the cost-effectiveness of CCT in the context of depression are largely missing. Health economic evaluation (HEE) allows to inform decision makers with evidence-based insights about how to spend limited available (financial) resources in the most efficient way. HEE studies constitute a crucial step in the implementation of a new intervention in clinical practice. Approaching preventive measures for depression such as CCT from an HEE perspective is informative to health policy, fostering optimal use of health expenditures. The aim of this paper was to inform and guide researchers during the phase of designing HEE studies in the context of CCT for depression. A clear view of CCT’s cost-effectiveness is paramount for its clinical implementation.

## Introduction

Depression is a leading cause of disability worldwide, with devastating effects on individual well-being and functioning. Globally, >5% of the population is affected by depression [1]. In terms of 12-month and lifetime prevalence of major depressive disorder (MDD), a recent American study reported 10.4% and 20.6% prevalence rates, respectively [2].

Available and effective psychological treatments for depression are expensive and typically require extensive expertise, resulting in only a small portion of individuals with depression being able to profit from those. Despite significant investments in pharmacological and psychological interventions in the past decades, low-income and middle-income countries still have limited accessibility to treatment, resulting in 75% of people with mental health disorders worldwide not receiving adequate care [1].

The severity of depression, combined with the low accessibility of treatments, leads to important personal and societal costs [3,4]. In 2006, Kessler et al [5] estimated, among a sample of US workers, that a single episode of MDD was associated with an average of >5 weeks of lost productivity per worker, resulting in an annual capital loss of >US $36 billion to employers. As such, there is an unmet need for widely accessible and cost-effective treatments for depression. Recently, the use of various technologies to deliver treatments has shown potential, especially in its ability to reach a large proportion of individuals who would otherwise not have access to psychological interventions [3,6]. One such intervention that shows substantial promise is cognitive control training (CCT) [7,8].

This paper discusses the current state of the art regarding the role of cognitive control impairments as a risk factor for depression and, consequently, the relevance of CCT in the prevention of depression. Building on recent meta-analyses suggesting a need for high-quality and adequately powered health economic evaluation (HEE) studies [9], we argue that adopting a health economic perspective is paramount to comprehensively evaluate the dissemination potential of CCT for depression in an evidence-based way. For this purpose, we have provided theoretical and practical guidelines about how to design HEE studies that allow to address this specific question.

## Cognitive Control Deficits as a Depressive Risk Factor

Identifying risk factors associated with recurrent depression has been the focus of extensive studies in the past decades, in which cognitive processes have shown to play an important role. Specifically, cognitive control—referring to the ability to flexibly adapt thoughts and behavior to achieve one’s goals [10]—has been identified as an important cognitive risk factor in the context of depression [11]. That is, cognitive control deficits have been observed in individuals with MDD [12] and often persist following remission [13], placing them at risk for the recurrence of depression [14].

Individuals with remitted depression show, among other things, working memory impairments, diminished processing speed and attention, and executive functioning impairments. These deficits have major consequences on an individual’s daily functioning and provide an important source of disability [15]. One of the main causes of costs associated with depression is loss of productivity owing to multiple or prolonged periods of sick leave or unemployment, where cognitive complaints interfere with occupational activities [16].

Previous studies suggest a central role for rumination in linking cognitive control deficits to risk for recurrent depression. Rumination can be defined as a recurrent process of perseverative negative thinking about feelings, problems, or disturbing experiences [17,18]. Elevated rumination, a key risk factor for depression, is associated with poor rates of recovery following pharmacological treatment for depression and slow response to psychotherapy [19,20]. In this context, it has been suggested that cognitive control is essential to be able to disengage from negative self-referential thoughts upon confrontation with stressors [21] or to inhibit habitual ruminative responses [22], where failure to do so may result in prolonged exposure to negative thoughts and affect. Consistent with this hypothesis, previous studies suggest that cognitive control deficits predict future increases in depressive symptomatology in patients with remitted depression, a relationship that appears to be mediated by rumination [23].

Although current pharmacological or psychological treatments have proven to be effective in decreasing depressive symptomatology, existing interventions typically do not target cognitive impairments directly. Moreover, there is a problem of recurrence of depression after initial treatment, where perturbations at the level of cognitive functioning show a positive association with the number of previous depressive episodes [24]. As such, it has been suggested that cognitive control deficits reflect increased vulnerability to recurrent depression [14]. Together, these findings suggest the need for innovative interventions aimed at reducing these specific risk factors.

## CCT for Depression

A particularly promising approach is CCT—a computerized psychological intervention during which operations and mental exercises aimed at improving cognitive control are performed [8]. Within this context, cognitive training has shown to be a useful intervention to reduce vulnerability to depression [25-27].

Among the multiple CCT tasks used within the context of depression, the effects of the adaptive paced auditory serial addition task (aPASAT [8]; for a review, please see the paper by Koster et al [7]) have been the most intensively studied. During this computer task, participants are presented with digits in a sequential fashion. They are asked to provide the sum of the 2 last heard digits. The aPASAT tailors the exercise based on individual performance by modifying the intertrial interval following every 4 consecutive correct (−100 milliseconds) or incorrect (+100 milliseconds) responses. Within the context of this task, individuals are continuously required to update information in working memory while preventing interference from previous responses.

Several studies suggest the beneficial effects of aPASAT training on depressive symptomatology. For instance, Siegle et al [8] investigated the impact of CCT on the neurobiological and cognitive mechanisms underlying depression in a sample of patients with clinical depression (N=31). In the first pilot study, the authors combined treatment as usual (TAU) with 6 sessions of aPASAT training and an attention training session during a period of 2 weeks. They observed beneficial effects of CCT on rumination and level of depressive symptomatology, in comparison with the TAU control group. In an extended sample (N=43), Siegle et al [28] replicated the previous positive effects of CCT on rumination among patients with MDD and observed a decreased need for clinical care at 1-year follow-up, providing the first evidence for the long-term benefits of CCT. The clinical potential of cognitive training has been confirmed in other studies reporting the beneficial effects of CCT in patients with MDD (eg, the studies by Brunoni et al [29], Iacoviello et al [30], Morimoto et al [31], and Sommer and Plewnia [32], but see the studies by Ferrari [33] and Moshier and Otto [34]).

Importantly, the beneficial effects of aPASAT training on depressive symptomatology were also observed in individuals at risk for (recurrence of) depression. For instance, in a sample of 68 individuals with remitted depression, Hoorelbeke and Koster [35] observed beneficial effects of 10 sessions of aPASAT training on rumination and depressive symptomatology compared with an active control condition. Similarly, in a recent randomized controlled trial (N=92 individuals with remitted depression) in which CCT was combined with an intensive experience sampling procedure, Hoorelbeke et al [36] observed beneficial effects of CCT on the deployment of rumination in daily life. Moreover, compared to an active control condition, CCT reduced the risk for recurrence of depression in individuals with remitted depression, as shown by lower recurrence rates in a period of one year following training [37].

CCT has shown interesting preventive effects in the context of depression. Consistent with this finding, a recent meta-analysis suggested small to medium effects of aPASAT training on depressive symptomatology and rumination, both immediately after training and at follow-up [38]. Interestingly, the authors explored the clinical relevance of their results by converting the pooled effect sizes into number needed to treat (NNT; ie, the number of patients who would need to be treated by an intervention to observe improvement in 1 patient). The results suggest that a similar number of patients should be treated with aPASAT training (NNT=6.15) to approximate the effects of monotherapeutic pharmacological (NNT=5.95) or psychotherapeutic interventions (NNT=7.13 [39]). Despite these encouraging findings, several challenges remain, such as identifying the precise mechanisms of transfer and the optimal parameters of administration (eg, training dosage and use of therapist-supported vs unguided delivery), which have mostly been left unexplored so far [40] (but see the study by Launder et al [25]).

Taken together, CCT is a highly promising preventive intervention for recurrent depression. Given that recent findings suggest that CCT may reduce the risk for recurrence of depression [37], CCT is also likely to influence related factors, including work absenteeism and costs associated with depression. By targeting underlying vulnerability factors that are currently not targeted by traditional interventions for depression, CCT has multiple characteristics that make it an interesting choice from a clinical perspective: (1) it could be relatively low cost intensive for patients; (2) it is highly accessible as it can be provided as a web-based intervention; (3) it is easily disseminated [7]; (4) it is a relatively inexpensive intervention, especially when provided on a large scale; and (5) compared with pharmacological treatments, limited side effects have been reported for CCT [26].

## Introducing a Health Economic Perspective

In recent decades, the importance of investing in health has been widely emphasized, resulting in a strong emphasis on the efficient use of budgets (eg, encouraging investments in preventive efforts) [41]*.* Therefore, policy makers have been strongly encouraged to opt for the most efficient interventions to ensure population health [42]. On the basis of effectiveness studies, digital health interventions seem to hold great promise as preventive interventions for depression [6,43-45]. In particular, CCT is a potentially scalable preventive intervention for recurrent depression that would suit web-based dissemination [38,40]. However, it is clear that a sole focus on the efficacy and effectiveness of CCT is insufficient for policy making. This would require HEE studies, examining the value for money of CCT compared with alternative strategies, aiming to inform different stakeholders (eg, decision makers) with evidence-based insights [42].

Health economics and HEE can be defined as “the study of how scarce resources are allocated among alternative uses for the care of sickness and the promotion, maintenance and improvement of health, including the study of how healthcare and health-related services, their costs and benefits, and health itself are distributed among individuals and groups in society” [46]. Multimedia Appendix 1 [42,46-51] provides an overview of the key health economic terms relevant to this paper.

Although multiple studies have explored the cost-effectiveness of other digital health interventions for depression [52-54] (for a recent meta-analysis, refer to the paper by Rohrbach et al [9]), the cost-effectiveness of CCT remains to be evaluated. If we aim to provide clear information to policy makers and governments, costs and health outcomes of CCT should be explored, compared with the available alternatives. As such, HEE forms a crucial step for the implementation of CCT.

To ensure that HEEs are methodologically correct, identifiable, interpretable, and useful for decision-making, several elements need to be considered, accounted for, and reported. The aim of this paper was to introduce HEE in the context of CCT and to discuss key considerations when planning an HEE. A focus will be placed on key methodological concepts when preparing an HEE of CCT for depression. This may guide researchers during the design phase of future studies. Multimedia Appendix 2 [55-68] provides an overview of these concepts and their translation to the context of CCT.

## Evaluating the Cost-Effectiveness of CCT

### Overview

HEE focuses on the comparative analysis of ≥2 alternative courses (intervention vs comparator) of action in terms of both their costs and effects. In the context of CCT, CCT can be considered as the intervention, whereas the comparator may be a waiting list condition, TAU, or a condition in which participants complete an alternative training procedure, with or without TAU. Overall, 4 main types of full HEE exist: cost-minimization analysis, cost-benefit analysis, cost-effectiveness analysis, and cost-utility analysis (CUA; for the definitions of these constructs, refer to Multimedia Appendix 1 [48]). As one of the most common HEEs used, CUA allows for health-related quality of life (HRQoL) adjustments to a given set of treatment outcomes, while simultaneously providing a generic outcome measure for comparison of costs and effects between different intervention strategies. Therefore, this paper specifically focused on this type of economic evaluation.

### HEE—Health Effects and Costs

As previously mentioned, in an HEE, both costs and effects are considered. Main *cost categories* include direct and indirect costs. Direct costs are those directly associated with the disease or condition (in this case, depression) that is considered. They can be divided into direct medical costs (eg, psychologist visit and hospitalization) and direct nonmedical costs (eg, transportation costs). Indirect costs include those associated with productivity losses owing to the disease or condition that is considered (Multimedia Appendix 1 [49]).

The types of costs that are considered in an HEE depend on the *perspective of the analysis*. Every time an economic question is asked, it is crucial to carefully consider the analytic viewpoint. Commonly used perspectives include (1) patient perspective (only considering the costs for the patient), (2) health insurance perspective (considering the costs for the health insurer; ie, direct medical costs), (3) payer perspective (ie, the patient and the health insurer), and (4) societal perspective (considering direct costs and indirect costs). Importantly, decision makers must be informed about the viewpoint that has been taken in the HEE.

The *effects of the intervention* may be measured in natural units (eg, avoided recurrent episode of depression, avoided hospitalization, avoided complications, and avoided psychotherapy sessions) or in utilities. In a CUA, the effects are typically expressed in terms of quality-adjusted life years (QALYs; Multimedia Appendix 1). QALYs are a standardized measure reflecting the extent to which interventions improve HRQoL [69]. QALYs are calculated by multiplying a utility for a given condition (HRQoL weight) by the time an individual experiences the condition. These utilities are often derived from generic questionnaires such as the Short Form Health Survey (SF-36) or EuroQol–5 Dimension. The SF-36 is a 36-item patient-reported survey allowing to measure health status [55,56]. Similarly, the EuroQol–5 Dimension is a widely used measure of HRQoL, allowing to estimate utilities to calculate QALYs [57].

QALYs are a recognized metric for evaluating treatments, allowing the comparison of intervention alternatives for different disorders. However, at the same time, the suitability of QALYs to assess changes in mental health has been questioned [69,70]. That is, measurement tools used to estimate QALYs typically contain a limited focus on (specific aspects of) mental health [70]. Therefore, measures such as the SF-36 have been criticized in terms of the extent to which they allow to capture the heterogeneous nature of depression. As depression is a complex construct to capture [66], the key challenges to obtaining QALYs include describing this state and valuing it (eg, in terms of the morbidity and quality of life associated with living in this health state) [71]. In this context, authors have highlighted the need for new conceptualizations of the QALYs, with the goal of basing it on a valid and comprehensive model of quality of life specific to mental illness. This would require an instrument that is more sensitive to detect expected changes in this context [72,73].

To overcome these challenges and increase efficiency in measurement, it has been suggested to combine QALYs with other outcomes. As such, we would advise the reader to consider different kinds of measurements, allowing a multimethod evaluation of cost-effectiveness (refer to Multimedia Appendix 2 for an overview of relevant outcome measures in the context of CCT and how these can be operationalized). In this context, patient-reported outcomes (PRO) and PRO measures (PROMs) are increasingly used. PROs contain information from patients about their quality of life, their own health, and the functional status related to their treatment or the health care they receive. PROMs are the instruments or tools that are used to measure PROs [74]. Considering both QALYs and PROMs in HEE could facilitate decision-making, improve the quality of health care, and stimulate improvements in services, essentially by enabling the comparison of providers’ performances [74,75].

### HEE—Outcome

The outcome of a CUA is usually expressed as the incremental cost-utility ratio (ICUR; Multimedia Appendix 1). This is calculated by dividing the difference in costs (incremental costs) between the intervention and the comparator by the difference in their effects (incremental effects). The ICUR results in the following outcome: € per QALY. An ICUR as such does not allow to draw conclusions about whether an intervention can be considered as cost-effective.

To determine the cost-effectiveness of an intervention, a reference value above which the intervention is considered as not cost-effective can be used. Reference values can be based on a fixed amount threshold or calculated based on gross domestic product (GDP) per capita. This is the case in most low-income and middle-income countries. The World Health Organization promotes a threshold defined as 1 to 3 times the national annual GDP per capita as a guide for determining the cost-effectiveness of health interventions. We refer interested readers to the papers by Kazibwe et al [76] and Woods et al [77] for a more detailed discussion about this topic.

The reference values used typically differ among countries. For example, in France, no explicit threshold is applied [78]. This is similar to Belgium [48], but a threshold of 1 time the GDP per capita, representing a value of €35,000 (US $38,080) to €40,000 (US $43,520) per QALY, is often used in HEEs [79-81]. In the Netherlands, the threshold is up to €80,000 (US $87,039) per QALY [82], whereas in Spain, this is typically €30,000 (US $32,640) per QALY [83]. The National Institute for Health and Care Excellence considers a threshold ranging between £20,000 (US $25,488) and £30,000 (US $38,232) for England and Wales [84]. In the United States, a threshold between US $50,000 and US $100,000 is still often used by researchers, insurers, and public and private policy makers, but there is a debate about whether the threshold should be adjusted to inflation [85,86]. In India, researchers often use the World Health Organization per capita GDP as a cost-effectiveness threshold [87].

### HEE—Sources When Thinking About an HEE

Guidelines may be useful for researchers when planning an HEE. Well-known reporting guidelines include the Consolidated Health Economic Evaluation Reporting Standards statement that was recently updated, providing resources reflecting the most recent developments in HEE methods [88]. This checklist includes 28 items and is primarily intended to guide researchers when reporting economic evaluations for peer-reviewed journals and peer reviewers and editors assessing these manuscripts for publication. Taking such guidelines into account in the phase of planning an HEE study is likely to add to the quality of the design.

Another checklist that has been widely used in this context is the Consensus Health Economic Criteria checklist. It has been designed for assessment of the methodological quality of economic evaluations in systematic reviews [89]. These contain items that need to be included as good practice when reporting results of an HEE. Many countries have also developed specific national guidelines. For instance, Sharma et al [90] identified 31 national HEE guidelines, published between 1997 and August 2020.

In addition to the guidelines mentioned previously, published HEEs within the field of CCT and depression prevention also provide valuable sources of information. We advise the reader to search appropriate literature for what is already known regarding the cost-effectiveness of a given intervention, preferentially by referring to peer-reviewed and evidence-based sources. To the best of our knowledge, there are currently no studies examining the cost-effectiveness of CCT for depression. Although not specific for CCT, a recent meta-analysis suggests that digital health interventions are slightly more cost-effective than usual care [9]. Consistent with previous studies [45,91,92], guided digital health interventions outperformed unguided interventions [9]. A factor that may have contributed to this is that most unguided digital health interventions typically do not compensate for the lack of therapist support by including additional content or technological features (eg, to support adherence) [93]. In this context, it should be noted that studies exploring the effects of CCT on indicators of vulnerability to depression strongly differ in the extent to which they have been delivered as a therapist-assisted or unguided web-based intervention. This, in addition to other factors related to the delivery mode of CCT [40], remains as a parameter that requires further investigation and should be taken into account when considering HEE of CCT.

Overall, the current paucity of research on HEE of CCT for depression suggests the need for future CCT studies to include a focus on HEE. To advance such research in the context of interventions for depression, standardization of studies in terms of measurement and analytic approaches is paramount [9]. For this purpose, Multimedia Appendix 2 provides an overview of methodological questions to consider when setting up such studies. In addition, for an overview of the optimal procedures to perform HEE (ie, data analysis), including step-by-step guidelines about how to interpret the results of such HEE, we refer to Briggs et al [67].

Several challenges related to performing HEE should also be considered. For instance, HEE outcomes will always be context specific and country specific (eg, an intervention that is cost-effective in Belgium might not be cost-effective in Portugal). In addition, findings may also differ depending on the perspective taken. For instance, a recent meta-analysis found the cost-effectiveness of digital health interventions to be moderated by the economic perspective used [9]. Studies using a societal perspective yielded a relatively lower incremental net monetary benefit (Multimedia Appendix 1) than studies adopting a health care perspective, suggesting that the cost-effectiveness of internet interventions compared with control conditions cannot be assumed when maintaining a societal perspective [9].

Depending on the economic perspective taken, additional resources may be available. For instance, Powell and Torous [94] recently proposed a patient-centered method for estimating the economic value of clinical improvement following the use of digital health interventions. In particular, the authors suggest that an estimate of the economic value of clinical response to digital health interventions can be obtained by multiplying the country-specific willingness-to-pay (WTP) threshold per QALY, impact of the mental health condition in terms of QALYs, engagement level of digital health intervention users, effect size of the intervention (eg, in terms of percentage of improvement in symptoms), and duration of the impact of the intervention.

Extending the nation-specific example provided by Powell and Torous [94] to the context of CCT for depression, the economic value of aPASAT training for depression is estimated at US $555.61 per individual treated in the United States. That is, following Powell and Torous [94], for the United States, we rely on WTP threshold values of US $175,000 per QALY and 0.159 QALYs lost per year of depression. We combine these estimates with data obtained from a recent study during which aPASAT training was used as a web-based digital health intervention targeting repetitive negative thinking [95]. A total of 382 participants completed the baseline assessment of this study, of which 152 reported a level of depressive symptoms of at least moderate severity (≥14) based on the Depression Anxiety and Stress Scales [96]. For this example, we consider these scores as potentially indicative for the presence of a depressive episode. Overall, 97 (64%) of the participants with elevated depressive symptoms completed the web-based intervention. Of the latter group, posttraining and follow-up data were available for 90 individuals, suggesting a median reduction in level of depressive symptoms of 33% and 39% immediately following the intervention and at 1 month follow-up, respectively. Using these parameters, we estimate the economic value of aPASAT training for depression as follows: US $175,000 per QALY × 0.159 QALYs lost per year of depression × 64% completing the intervention × 39% reduction in symptoms × 0.08 years of improvement (at least until 1 month follow-up)=US $555.61. This value could be compared with values obtained for other interventions and costs related to the use of the intervention (eg, subscription fees to obtain access to the digital health intervention) or be used to investigate the impact of country effects and context effects (eg, WTP threshold used).

Although informative to the patient, limitations of such methods include the imprecise nature of the estimation, owing to the input parameters used. For instance, in the example provided above, adherence and response rates were based on 1 study that relied on a convenience sample, in which evaluation of effects was limited to 1 month follow-up [95]. It is possible that the context in which CCT was provided offers an overestimation of adherence rates and, related to this, economical gains. Vice versa, previous research suggests that effects of CCT likely extend beyond the duration included in this example [35]. Other important limitations of this approach include the exclusive focus on the patient perspective and the lack of consideration of indirect benefits of the intervention (eg, increased productivity in a professional context). Such information can only be obtained from HEE studies designed to address the question of cost-effectiveness.

In this context, it is recommended for clinical trials in this area to systematically adopt measures that allow HEE based on input from various stakeholders. As CCT can be relatively inexpensive on a sufficiently large scale, we recommend researchers to cooperate with all the relevant stakeholders including patients, clinicians, health insurance companies, and policy makers. In addition, health economic researchers need to educate stakeholders about the relevance of cost-effectiveness analysis in policy and practice, how such analyses are performed, what uncertainties can be involved, and how the results should be interpreted. In addition to accurate communication with stakeholders, a clear view about CCT’s cost-effectiveness is paramount to its clinical implementation.

### HEE—Dealing With Uncertainty

It is worth noting that HEEs are frequently characterized by a certain degree of uncertainty or methodological considerations related to input parameters (eg, utilities and costs). This uncertainty can be addressed by performing 1-way sensitivity analyses and probabilistic sensitivity analyses (for more information, refer to Drummond et al [42]).

As noted previously, HEE serves evidence-based decision-making processes in health care. As such, we recommend that researchers include an HEE in their studies as a crucial step toward implementation. More precisely, information about the cost-effectiveness of CCT for depression will provide an extra piece of evidence that can be considered as being highly relevant for different stakeholders (eg, researchers, clinicians, and policy makers). However, the accuracy of such evidence is highly dependent on the quality of HEE methodological approaches used. This requires thorough reflection about methodological choices when planning future studies, which may be guided by the tools provided previously.

## Conclusions

Previous studies suggest the potential of CCT to be used as a scalable and effective preventive intervention for recurrent depression. Although previous studies have mainly focused on the effects of CCT in terms of cognitive and emotional transfer, so far, little attention has been given to the cost-benefit ratio in this context. As such, there is a strong need for investigation of the cost-effectiveness of CCT. This paper provided guidelines about how future studies can address this gap in the literature as a means to influence health care policy, paving the way toward clinical implementation of CCT.

## Appendix

Multimedia Appendix 1 Key health economic constructs and definitions of basic concepts.

Multimedia Appendix 2 Key considerations when designing health economic studies in the context of cognitive control training for depression.

## Abbreviations

aPASAT: adaptive paced auditory serial addition task
CCT: cognitive control training
CUA: cost-utility analysis
GDP: gross domestic product
HEE: health economic evaluation
HRQoL: health-related quality of life
ICUR: incremental cost-utility ratio
MDD: major depressive disorder
NNT: number needed to treat
PRO: patient-reported outcome
PROM: patient-reported outcome measure
QALY: quality-adjusted life year
SF-36: Short Form Health Survey
TAU: treatment as usual
WTP: willingness-to-pay
