# Supplementary material for: Health Economic Evaluation of Cognitive Control Training for Depression: Key Considerations
Source: JMIR Ment Health. 2023 Aug 18;10:e44679. doi: 10.2196/44679 (PMC10474514; doi:10.2196/44679)
Supplement: Multimedia Appendix 1 [file mental_v10i1e44679_app1.pdf]

Multimedia Appendix 1. Key health economic constructs and definitions of basic concepts.

**Key health economic term**	**Definition**
CBA[a]	CBA in health care is a comparison of interventions and their consequences that expresses all gains (costs and resulting benefits, including health outcomes), most of the time in monetary terms (or common units); CBA aims to evaluate to what extent a judgment or an objective should be pursued, enabling ≥2 treatment alternatives to be compared.
CEA[b]	CEA focuses on how to reach a stated objective defined in advance, and that will not be questioned. CEA evaluates the effectiveness of ≥2 treatments relative to their cost. The aim of the decision maker when assessing a new intervention is to maximize outcomes and minimize opportunity costs. CEA is the method used to measure these outcomes [47].
CMA[c]	In CMA, when comparisons are made between interventions, it is made only based on the resource costs, as the consequences of completing those interventions are identical. All the costs of the alternative interventions, which are known or assumed to have an equivalent medical effect, are compared. This includes the costs of managing any consequences of the interventions. The goal of CMA is to determine the least expensive way of achieving the same outcome. CMA can be used to determine which of the treatment alternatives produces the least expensive way of reaching a specific health outcome for a specific population [47].
CUA[d]	CUA is a form of CEA in which benefits are measured in terms of a utility measure such as QALY[e], allowing a generic outcome measure for the comparison of costs and effects between different intervention strategies [48]. For example, it is a useful way to compare different programs across different treatment areas.
DALY[f]	DALY is a generic measure of disease burden and health effect

	in a population. It can be used in CEA as an alternative of the QALY. This measure considers 2 components of disease burden—morbidity and mortality. Morbidity is related to the time spent in a health state characterized by a lowered quality of life owing to disability. Mortality comes into the equation when illness is associated with premature death. Concretely, health gains can be expressed as a reduction in terms of disease burden (eg, how much a health care intervention such as CCT[g] reduces the number of DALY in the population) [47].
Direct costs	Costs are expenses directly associated with the disease or condition under consideration. They can be divided into direct medical costs (eg, psychologist visit and hospitalization) and direct nonmedical costs (eg, transportation costs) [49].
HEE[h]	The study of how scarce resources are allocated among alternative uses for the care of sickness and the promotion, maintenance, and improvement of health, including the study of how health care and health-related services, their costs and benefits, and health itself are distributed among individuals and groups in society [46]. It is the comparative analysis of ≥2 alternative courses (intervention vs comparator) of action in terms of both their costs and effects [42].
HRQoL[i]	Individual or group's perceived physical and mental health over time [50].
ICER[j]	ICER is a summary measure representing the economic value of an intervention, compared with an alternative (comparator). It is usually the main output or result of an economic evaluation. An ICER is obtained by dividing the difference between the total costs of the 2 interventions (incremental cost) by the difference in the chosen measure of health outcome or effect (incremental effect) to provide a ratio of *extra cost per extra unit of health effect* for the more expensive therapy versus the alternative. ICERs reported in economic evaluations are compared with a predetermined threshold (refer to WTP[k] threshold) to decide

	whether choosing the new intervention is an efficient use of resources [47].
ICUR[l]	ICUR is calculated by dividing the difference in total costs between the 2 alternatives over the same specific time frame by the difference in the reported QALYs for each. ICUR gives the cost per additional QALY gained because of the intervention over its comparator. Many studies use the terms ICUR and ICER interchangeably. The ICUR (or ICER) will be compared with the WTP threshold.
Indirect costs	Costs associated with productivity losses due to the disease or condition that is considered [49].
Natural unit	Outcomes expressed as natural units (eg, days of absence, avoidance of the recurrence of depression, experienced residual symptomatology, repetitive negative thinking, avoided days of hospitalization or other indicators of consumption of health care services).
NMB[m]	NMB is calculated using the following formula: (incremental benefit × threshold) – incremental cost. It measures the difference in NMB between alternative interventions. A positive incremental NMB indicates that the intervention is cost-effective compared with the alternative at the given WTP threshold. Thus, the cost to derive the benefit is less than the maximum amount that the decision maker would be willing to pay for this benefit [47].
Perspective	The perspective of the analysis is the point of view from which an analysis is conducted. Choice of perspective determines the type of costs that are considered in the analyses and the stakeholders taken into account. Commonly used perspectives include the (1) patient perspective (only considering the costs for the patient); (2) health insurance perspective (considering the costs for the health insurer, ie, direct medical costs); (3) payer perspective (ie, the patient and the health insurer); and (4) societal perspective (considering direct costs and indirect costs).

	Importantly, decision makers must be informed about the viewpoint that has been taken in the HEE.
QALY	QALY is a summary outcome measure used to quantify the effectiveness of a particular intervention. As the benefits of different interventions are multidimensional, QALYs have been designed to combine the impact of gains in quality of life and quantity of life (ie, life expectancy) associated with an intervention. More specifically, QALYs are based on utilities, which are valuations of HRQoL measured on a scale in which full health is valued as 1 and death as 0. These valuations are multiplied by the duration of time (in years) that an individual spends in a health state with that specific utility score, and aggregate QALYs are then summed over the individual's projected lifetime (or other period corresponding to the time horizon of the analysis). QALYs are recommended by NICE[n] as a preferred measure of health outcome for use in technology appraisals [47]. In a CUA, the effects are typically expressed in QALYs.
Time horizon	Time horizon is the duration over which health outcomes and costs are calculated. The choice of time horizon is an important decision for economic modeling and depends on the nature of the disease and intervention under consideration and the purpose of the analysis. Longer time horizons are applicable to chronic conditions associated with ongoing medical management, rather than a cure. A lifetime horizon is preferred by UK NICE; however, it may be useful in sensitivity analysis to rely on intermediate time horizons of 5 to 10 years, for which there may be more robust data [51]. A shorter time horizon may be appropriate for some acute conditions, for which long-term consequences are less important. The same time horizon should be used for both costs and health outcomes.
WTP threshold	WTP threshold is the maximum amount a decision maker is willing to pay for a unit of health outcome. If the cost-

	effectiveness (ICER) of a new intervention (compared with a relevant comparator) is estimated to be below this threshold, then (other things being equal), it is likely that the decision maker will recommend the new treatment [47].

[a]CBA: cost-benefit analysis.

[b]CEA: cost-effectiveness analysis.

[c]CMA: cost-minimization analysis.

[d]CUA: cost-utility analysis.

[e]QALY: quality-adjusted life year.

[f]DALY: disability-adjusted life year

[g]CCT: cognitive control training.

[h]HEE: health economic evaluation.

[i]HRQoL: health-related quality of life.

[j]ICER: incremental cost-effectiveness ratio.

[k]WTP: willingness to pay.

[l]ICUR: incremental cost-utility ratio.

[m]NMB: net monetary benefit.

[n]NICE: National Institute for Health and Care Excellence.
