# Supplementary material for: Health Economic Evaluation of Cognitive Control Training for Depression: Key Considerations
Source: JMIR Ment Health. 2023 Aug 18;10:e44679. doi: 10.2196/44679 (PMC10474514; doi:10.2196/44679)
Supplement: Multimedia Appendix 2 [file mental_v10i1e44679_app2.pdf]

Multimedia Appendix 2. Key considerations when designing health economic studies (HEE) in the context of cognitive control training (CCT) for depression.

Guidance for reporting	Application in the context of CCT for depression
Comparators: Describe interventions being compared and provide justification	• Depending on the scope of the study, select relevant comparators, taking the method of delivery (eg, therapist supported) into account; for instance, comparison of a TAU[a] + CCT condition with a TAU condition, or comparison of a psychotherapy + CCT condition with ADM[b] + CCT condition, and so on.
Perspective: State the perspective(s) adopted by the study and provide justification	• The societal perspective may be preferred, given that depression renders both direct and indirect costs (eg, productivity losses). Other perspectives can also be relevant. For instance, the health insurance perspective will be preferred when the aim is to inform the health insurer.
Time horizon: State the time horizon for the study and provide justification	• Time horizon should be carefully chosen, as it must be sufficiently long to capture the consequences likely to be associated with depression. Specific research questions might warrant focusing on short-term versus long-term consequences:<br>o Short term with a treatment perspective: when investigating the immediate effects of the intervention in terms of cost-effectiveness (eg, when the focus is on augmenting the effects of existing treatments), it may be sufficient to examine treatment effects immediately after training and following a brief follow-up period.<br>o Long term with a preventive perspective: the investigation of preventive effects of CCT typically requires the use of longer follow-up periods (eg, ranging from months to several years).

	• Given the long-term benefits of studying depression, comprehensive HEE might require even longer periods, which may involve simulating long-term effects. However, note that when considering such a long-term perspective, it is crucial to be aware that certain assumptions are required, which impacts the accuracy of the findings. This can be handled by performing probabilistic sensitivity analysis.
Selection of outcomes: Justify the measure of benefit(s) and harm(s)	• In addition to the inclusion of measures that allow the estimation of QALYs[c], outcomes can also be expressed as natural units (eg, days of absence, avoidance of the recurrence of depression, experienced residual symptomatology, repetitive negative thinking, avoided days of hospitalization, and other indicators of consumption of health care services).
Measurement of outcomes: Describe how outcomes used to capture benefit(s) and harm(s) were measured	• QALYs can be obtained using EQ-5D-5L or SF-36[d] [55-57].<br>• Direct and indirect costs related to depression (eg, productivity loss, days of absence, and consumption of health care services) can be assessed using questionnaires such as the WPAI[e] [58] and iMCQ[f] [59]. Recurrence of depression can be assessed using structured clinical interviews (eg, MINI[g]) [60]. Severity of (residual) depressive symptoms may be assessed using measures such as the RDQ[h] [61], BDI-2[i] [62], or PHQ-9[j] [63], whereas repetitive negative thinking (eg, rumination) could be assessed using measures such as the PTQ[k] [64] or RRS[l] [65]. Here, it should be noted that there is a large availability of measures of depression, which can vary in validity and reliability [66].<br>• Potential harm and side effects of the intervention

	need to be addressed.
Rationale and description of the model: If HEE modeling is used, provide justification and detailed description	• The choice of the HEE model depends on the time horizon. For HEE, there are multiple modeling options (refer to the study by Briggs et al [67]).<br>• In the context of depression, models that allow the capture of recurrent influences in a time-sensitive dynamic are preferred (eg, Markov models; for a detailed example in the context of depression, refer to the study by Lokkerbol et al [68]). Such decision analytic tools allow the extrapolation of effects on costs and health effects.<br>• Consider making the model publicly available, as this would allow accuracy checks and repeated use.
Analytics and assumptions	• Describe the choice of methods used for analyzing or statistically transforming data and those used for extrapolation. Analytics and assumptions crucially depend on the time horizon.<br>• A longer time horizon typically requires the use of assumptions as long-term follow-up data from, for instance, clinical trials are often not available. Therefore, clearly describing the assumptions that were used is crucial for transparency reasons.<br>• Describe methods to characterize any source of uncertainty in the analysis.<br>• When combining data from several RCTs[m] to extrapolate long-term treatment effects, multilevel models can be used to account for the different sources of data.<br>• To address the uncertainty related to certain input parameters, both 1-way and probabilistic sensitivity analyses should be used.

Approach to stakeholder engagement	• Describe approaches to engage patients, service recipients, or stakeholders (clinicians or payers). In the context of depression, be sure to engage with individuals with RMD[n], clinicians, and other stakeholders.

[a]TAU: treatment as usual.

[b]ADM: antidepressant medication.

[c]QALY: quality-adjusted life year.

[d]SF-36: Short Form Health Survey.

[e]WPAI: Work Productivity and Activity Impairment Questionnaire.

[f]IMCQ: Medical Consumption Questionnaire.

[g]MINI: Mini-International Neuropsychiatric Interview.

[h]RDQ: Remission from Depression Questionnaire.

[i]BDI-2: Beck Depression Inventory–2.

[j]PHQ-9: 9-item Patient-Health Questionnaire.

[k]PTQ: Perseverative Thinking Questionnaire.

[l]RRS: Ruminative Response Scale.

[m]RCT: randomized controlled trial.

[n]RMD: remitted depression.
